# Benefits of listening to a recording of euphoric joint music making in polydrug abusers

**DOI:** 10.3389/fnhum.2015.00300

**Published:** 2015-06-11

**Authors:** Thomas Hans Fritz, Marius Vogt, Annette Lederer, Lydia Schneider, Eira Fomicheva, Martha Schneider, Arno Villringer

**Affiliations:** ^1^Max Planck Institute for Human Cognitive and Brain SciencesLeipzig, Germany; ^2^Department of Nuclear Medicine, University of LeipzigLeipzig, Germany; ^3^Institute for Psychoacoustics and Electronic Music (IPEM)Gent, Belgium

**Keywords:** music therapy, agency (psychology), exercise, mentalizing, mood disorders, addiction

## Abstract

**Background and Aims**: Listening to music can have powerful physiological and therapeutic effects. Some essential features of the mental mechanism underlying beneficial effects of music are probably strong physiological and emotional associations with music created during the act of music making. Here we tested this hypothesis in a clinical population of polydrug abusers in rehabilitation listening to a previously performed act of physiologically and emotionally intense music making.

**Methods**: Psychological effects of listening to self-made music that was created in a previous musical feedback intervention were assessed. In this procedure, participants produced music with exercise machines (Jymmin) which modulate musical sounds.

**Results**: The data showed a positive effect of listening to the recording of joint music making on self-efficacy, mood, and a readiness to engage socially. Furthermore, the data showed the powerful influence of context on how the recording evoked psychological benefits. The effects of listening to the self-made music were only observable when participants listened to their own performance first; listening to a control music piece first caused effects to deteriorate. We observed a positive correlation between participants’ mood and their desire to engage in social activities with their former training partners after listening to the self-made music. This shows that the observed effects of listening to the recording of the single musical feedback intervention are influenced by participants recapitulating intense pleasant social interactions during the Jymmin intervention.

**Conclusions**: Listening to music that was the outcome of a previous musical feedback (Jymmin) intervention has beneficial psychological and probably social effects in patients that had suffered from polydrug addiction, increasing self-efficacy, mood, and a readiness to engage socially. These intervention effects, however, depend on the context in which the music recordings are presented.

Patients with drug-related disorders are known to have a substance abuse related malfunction of the reward system as a consequence of habituation to high levels of reward-mediating neurotransmitters, which deplete faster from the synaptic gap (Koob and Le Moal, 2005; Volkow et al., 2011). It has been shown that one consequence of this neurological malfunction is a diminished self-efficacy (the perception of having control over one’s life; Beck and Lindenmeyer, 1997).

Polydrug abusers also often exhibit antisocial behavior and become involved in criminal activity (Gandossy et al., 1980; Prichard and Payne, 2005). Polydrug abusers often have deficits in social skills, including the development of stable interpersonal relationships (Batra, 2011), and it has been described that patients in a drug clinic can take advantage of interventions fostering social skills (Hawkins et al., 1986; Wittchen, 2006). Making music is known to increase group commitment in therapeutical settings (Cassity, 1976; Henderson, 1983), and may be used to improve social competence (Gooding, 2011). It seems that polydrug abusers respond well to music therapy; as such, it is commonly used as a treatment in patients with substance-related disorders (Gallagher and Steele, 2002; Winkelman, 2003). For this purpose, several musical interventions have been described, including: rhythm activities (Cevasco et al., 2005), improvisation (Murphy, 1983), song-writing (Freed, 1987), lyric analysis, where participants label passages in musical lyrics with which they identify (Walker, 1995) or playing instruments (Miller, 1970).

A positive attitude to life can have a huge range of other psychological effects that are probably indirectly beneficial to the health and well-being of the individual (Scheier et al., 1989; Karademas, 2006). For therapeutic application it is therefore generally desirable to systematically evoke beneficial memories and associations that lead to a positive attitude. Music listening can evoke such positive and therapeutically potentially beneficial effects through strong emotional responses in music listeners (Sloboda, 1991; Jäncke, 2008), and accordingly music has also been shown to modulate physiological arousal, which has been regarded a basic dimension of emotion in a variety of dimensional emotion models (Husain et al., 2002).

Listening to a recording of one’s own musical performance, however, through association with the original performance has been shown to be even superior at evoking emotional and physiological experiences (Sutherland et al., 2009). On a motor level, listening to a previously practiced piano melody has been shown to lead to associations of the movements used to create the respective melodies (Lahav et al., 2005). Such newly created auditory-motor associations have been successfully used in therapy to enhance motor performance in patients (Sutherland et al., 2009), and passively listening to recordings of such musical auditory-motor associations have been shown to increase motor learning (Wan et al., 2011).

It is yet unclear if passively listening to recordings of previous performances also has clinically applicable psychological effects, such as those investigated in the current study with polydrug abusers. Here we used a memory induction method, stimulating previous motor and other physiological and psychological experiences during a musical feedback paradigm with Jymmin machines. This intervention allows participants to express themselves musically by exercising on fitness machines that have been modified to transform physical movements into musical sounds and thus allow music to be played interactively in a group. This combination of musical expression and extreme physiological arousal has been shown to create an intense musical and emotional experience that correlates with a decreased sense of exertion (Fritz et al., 2013a) and an enhanced mood (Fritz et al., 2013b).

The present study aimed to assess the psychological effects in polydrug abusers when presenting them a recording that participants had done 1 week before in an emotionally and physiologically intense Jymmin musical feedback intervention. We hypothesized that given the physiological and emotional associations with music created during the act of music making, listening to the recording would lead to greater psychological benefits in terms of increased mood and internal locus of control than listening to a similar piece of music that was not self-created.

## Methods

### Participants

Twenty-two participants (19 male), were tested within an age range of 20–47 in males (mean = 31.11) and 27–43 in females (mean = 37.5). Participants used the fitness machines in groups of three. None of the participants were professional body builders, musicians, or athletes. Clinical data showed that 77.7% of the participants consumed more than two drugs regularly during the pre-clinic period (polydrug use). 63% were involved in criminal activities and therefore incarcerated for between 1 and 96 months. The majority of the investigated population had been sentenced to jail at the time of intervention and were doing the rehabilitation program during their prison sentence (§35 in the German Controlled Substances Act). 48.1% of them had ADHD or related hyperkinetic disorders as a comorbid diagnosis.

Informed consent was obtained from all of the subjects and the experiment was conducted in accordance with the Declaration of Helsinki’s ethical principles for research involving humans. It conformed to internationally accepted policy statements regarding the use of human subjects and was approved by the ethics committee of the University of Leipzig.

### Experimental Design

The experiment included two conditions. In the first condition, participants listened to a recording of their previously performed interactive musical feedback session with Jymmin machines. Note that in the current manuscript we use the word Jymmin not in terms of a genre, but to describe the specific configuration used for the musical feedback system. In the second condition (control condition), they listened to a commercially available music piece—a commercial drum and bass track—that was similar in style to the Jymmin pieces. In order to enhance the ecological validity of the control condition, the control stimulus was selected from a variety of commercially available drum and bass tracks so that each participant listened to a different control excerpt. We controlled for the effects of sequence of condition by counterbalancing condition orders. Half of the participants listened to the Jymmin piece first (sequence one), followed by the control piece. The other half listened to the control piece first and then listened to the recording of their own music session (sequence two).

### Experimental Procedure

The participants were recruited from a drug rehabilitation clinic. The musical feedback session was recorded 5 days prior to the current experiment. In this music session, participants worked out on fitness machines (stomach trainer, stepper and cable lat pulldown) that were enhanced with sensors and a sound system to produce musical sounds through workout movement (Fritz et al., 2013a,b). Musical parameters that were modulated by the group were cutoff-filter and pitch of different tracks of a largely predefined musical piece. The music was performed in groups of three for 10 min. The participants had been shown how to do movements on the fitness machines in a sport physiologically correct way, and were briefly demonstrated the musical sounds each machine could create. They received the instruction: “Please use the fitness machine in a way that you are physically comfortable with”. When working out, participants created the musical piece in a group performance. This paradigm had previously been shown to be effective at evoking euphoric musical experiences through a combination of musical expression and physical exertion (for a detailed description, see Fritz et al., 2013a,b).

All participants filled out a questionnaire following both of the two conditions (see Section Experimental Design). No baseline measurements were conducted before presenting either of the two conditions. Participants filled out general information items on gender and age. The questionnaire contained the PANAS scale (Krohne et al., 1996), the internal vs. external locus of control short scale (Kovaleva, 2012), and a subscale of the Multidimensional Mood Questionnaire (MDMQ; Mehrdimensionaler Befindlichkeitsfragebogen; Steyer et al., 1997). The following seven self-designed items were presented after each experimental condition (“When I listen to the music piece, I have positive thoughts”, “I feel relaxed”, “I would like to perform another ‘Jymmin’ session”, “I am in the mood for exercise”, “I feel peaceful”, “I felt creatively inspired”, “I have negative thoughts”). Six additional self-constructed items were assessed after the Jymmin condition (“When I listen to the music piece, I want to engage in social activities with my former workout partners”, “I think of the ‘Jymmin’ session”, “I consider my former training partners to be interesting”, “I think of my former training partners”, “I consider my former training partners to be nice people”, “I want to perform another ‘Jymmin’ session with my former training partners”).

The combined questionnaire was filled out following each of the two conditions. Participants took a break of 5 min after the first condition in order to fill out the questionnaire. After that, they were immediately exposed to the second condition. After the second condition, they were asked to fill out the second questionnaire. All of the participants were given commercially available MP3 players with stereo headphones to listen to the previously recorded musical feedback sessions and the control music piece.

The PANAS scale assesses positive and negative affect using 20 items with a five-point Likert scale from 1 (“hardly or not at all”) to 5 (“extremely”). The 20 items consist of adjectives that describe a mental state (e.g., “active”, “interested”, or “nervous”). Participants should rate their current emotional state by choosing their most favorable answer. Within the test, positive and negative affects are interpreted as independent dimensions. The PANAS is a well-established measure of psychological well-being and has been proven valid and reliable, even though the independent two-factorial structure of negative and positive affect has been questioned (Krohne et al., 1996; Crawford and Henry, 2004). However, with an internal consistency (Cronbach’s Alpha) of *α* = 0.84 and *α* = 0.86 for negative vs. positive affect (Watson et al., 1988), it has good reliability.

The locus of control short-scale measures a self-regulation-variable with four items and a five-step Likert scale ranging from 1 (“does not apply”) to 5 (“fully applies”). The first and second items measure internal loci (e.g., “If I work hard, I’ll succeed.”), whereas the third and fourth assess external loci (e.g., “My plans are controlled by fate.”). The locus of control concept is a key variable in the social cognitive theory of personality (Rotter, 1954). An internal locus of control indicates the general expectation to have control over pleasant events in one’s life (e.g., social recognition, goal achievement) and to avoid unpleasant punishments (e.g., pain, hunger). The external locus of control is a stable belief that pleasant sources of reinforcement cannot be controlled by the individual (e.g., professional failure is unavoidable). A high internal locus of control correlates with the assumption that the individual is personally accountable for their own positive behavioral outcomes, whereas a high external locus goes along with the belief that behavioral outcomes are controlled by external sources such as coincidence or fate. The short-scale measure showed an estimated internal consistency that was sufficient (McDonald’s Omega (ω) varying between 0.71 and 0.73; Kovaleva, 2012).

The MDMQ is considered a mental state measurement of current mood (Steyer et al., 1997). The questionnaire contains three bipolar subscales (“good vs. bad mood”, “calmness vs. agitation” and “alertness vs. tiredness”) each with eight items. Each subscale consists of eight adjectives, of which four belong to the negative (e.g., “bad”, “uncomfortable”) and four to the positive (e.g., “well”, “satisfied”) pole. For our study, we only used the ”good” vs. “bad” mood subscale. The items are rated on a five-point Likert scale ranging from 1 (“not at all”) to 5 (“very”). Despite the short length of the measure (eight items), reliability is high with an internal consistency (Cronbach’s alpha) between *α* = 0.73 and *α* = 0.89 (Heinrichs and Nater, 2002).

### Data Analysis

The behavioral ordinal-scaled data were analyzed using SPSS 21 (IBM). Missing values were indicated in SPSS and not included in the analysis. In order to obtain mean scores for the subscales of the PANAS, locus of control and mood questionnaire, responses for each subscale were averaged. The PANAS consists of two subscales describing the participant’s positive and negative affect. Ten positive items, such as “powerful”, “interested”, and “excited” were averaged for the subscale of positive affect. Out of the remaining 10 negative items, e.g., “angry”, “ashamed”, and “hostile”, a mean score was formed indicating the negative affect. The first two items of the four-item locus of control scale measure internal locus, whereas the second half of the scale indicates external locus. For each subscale averages were formed. Items of the MDMQ belonging to the negative pole (e.g., “bad” or “uncomfortable”) were recoded before data analysis so that higher scores on the negative pole were related to good mood. All of these mean scores range from 1–5, reflecting the 1–5 Likert scale used to rate each item.

A Kruskal-Wallis H Test was performed to test the effect of condition order (listening to Jymmin music first, listening to control music first) on the mean scores of PANAS, locus of control and MDMQ. Condition order varied between subjects. Follow-up Mann-Whitney U Tests for independent samples were performed for each of the significant subscales to determine the direction of the effect. In order to correct for multiple comparisons the Bonferroni correction was used and a significant alpha level was set to 0.017. A Spearman’s correlation matrix was generated to explore correlation effects between self-designed items and scores of locus of control and MDMQ in the Jymmin condition.

## Results

**Figure 1:**
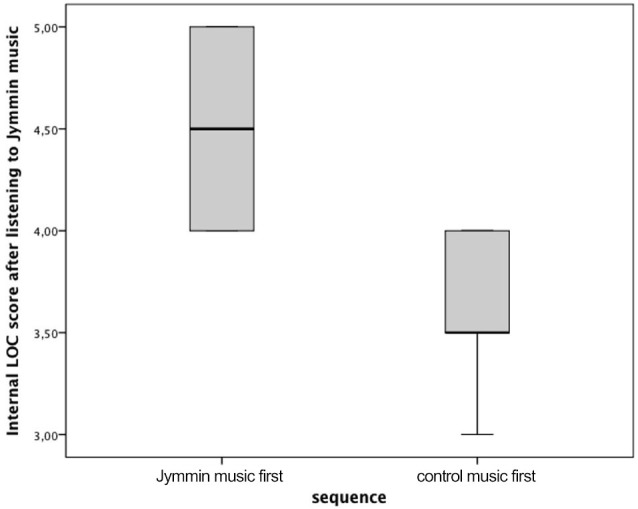
**The figure depicts scores on the internal locus of control scale after listening to a recording of the Jymmin performance either first or following a control music piece**. A Kruskal-Wallis H Test revealed a significant difference between medians of both sequences.

A Kruskal-Wallis H Test revealed that internal control in the Jymmin condition differed significantly between participants of sequence one (Jymmin song first) and sequence two (control song first), $\chi_{\mathrm{(1)}}^{2}$ = 6.599, *p* = 0.010. A follow up Mann-Whitney U Test for independent samples showed that the score of internal control was significantly higher in the Jymmin condition of sequence one (*Mdn* = 4.50) compared to the Jymmin condition in sequence two (*Mdn* = 3.50), *U* = 22, *Z* = −2.57, *p* = 0.010, *r* = −0.55 (see Figure 1). However, there were no significant differences in the score of internal control between the Jymmin and control music condition of sequence two.

**Figure 2:**
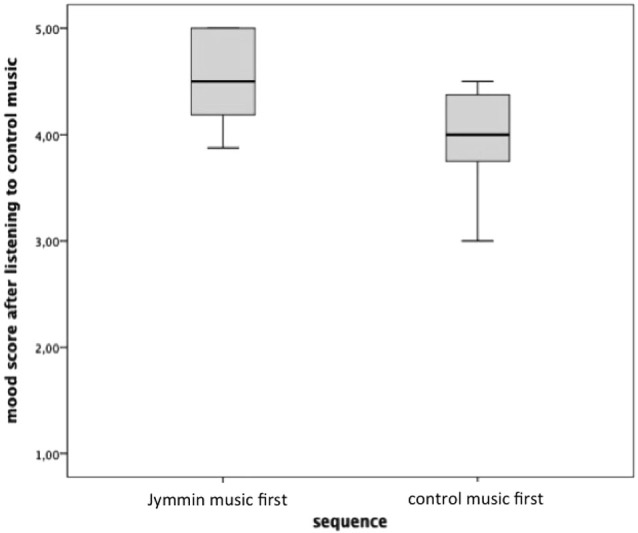
**The figure depicts scores on the MDMQ scale after listening to the control music piece, either first or following the Jymmin recording**. A Kruskal-Wallis H Test revealed a significant difference between medians of both sequences.

Furthermore, the Kruskal-Wallis H Test showed that mood differed significantly between participants of the first and second sequence in the control condition ($\chi_{\mathrm{(1)}}^{2}$ = 6.548, *p* = 0.010). A follow-up Mann-Whitney U Test for independent samples showed that participants of sequence one had an increased mood score (*Mdn* = 4.50) compared to participants of sequence two in the control condition (*Mdn* = 4.00), *U* = 21.5, *Z* = −2.56, *p* = 0.010, *r* = −0.55 (see Figure 2). However, participants of sequence one (*Mdn* = 4.56) and of sequence two (*Mdn* = 4.19) in the Jymmin condition, did not differ significantly in their mood scores ($\chi_{\mathrm{(1)}}^{2}$ = 4.421, *p* = 0.035).

A Kruskal-Wallis H Test revealed no significant differences with regard to the positive and negative affect (PANAS scale) of participants of the first and second sequence in the Jymmin condition ($\chi_{\mathrm{(1)}}^{2}$ = 3.297, *p* = 0.069 and $\chi_{\mathrm{(1)}}^{2}$ = 0.502, *p* = 0.478) in the control condition ($\chi_{\mathrm{(1)}}^{2}$ = 2.121, *p* = 0.145 and $\chi_{\mathrm{(1)}}^{2}$ = 4.805, *p* = 0.028).

A Mann-Whitney U Test for independent samples revealed significant differences between participants of both sequences with regard to the following dependent variable: participants of sequence one reported an increased desire to do sports in general (*Mdn* = 3.0) compared to participants in sequence two (*Mdn* = 2.5), *U =* 26.0, *Z* = −2.53, *p* = 0.012, *r* = −0.54, after listening to the Jymmin music piece.

**Figure 3:**
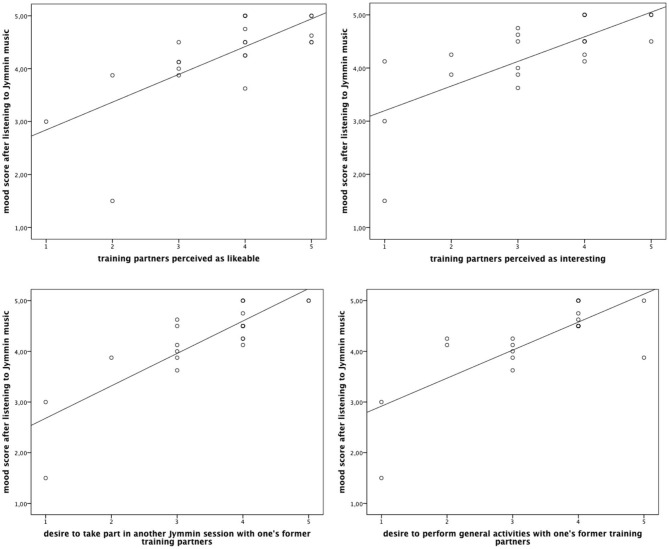
**The figure depicts Spearman’s correlations between participant’s mood (MDMQ score) after listening to a recording of the Jymmin performance and the readiness to engage socially (self-contructed items)**.

Spearman’s correlation matrices show that participants who felt more content, happy, and comfortable also reported to think about their training partner as a more likeable (*r_s_* = 0.722, *p* < 0.001) and interesting individual (*r_s_* = 0.702, *p* < 0.001). Additionally, an increased mood correlated with the desire to take part in another Jymmin session with the same training partners, *r_s_* = 0.774, *p* < 0.001, and the desire to perform general activities with one’s former training partners, *r_s_* = 0.695, *p* < 0.001 (see Figure 3).

**Figure 4:**
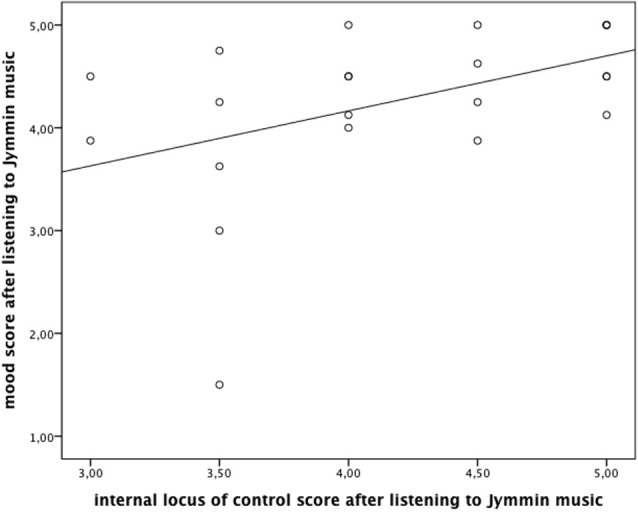
**The figure depicts Spearman’s correlations between participant’s mood (MDMQ score) and the internal locus of control after listening to the Jymmin recording**.

Spearman’s correlation matrices show that participants who after listening to their Jymmin recording had a better mood also reported a higher score on the internal locus of control scale (*r_s_* = 0.495, *p* < 0.019; see Figure 4).

## Discussion

The present study investigated the psychological benefits of passively listening to self-made recordings of a musical feedback intervention (Jymmin) in polydrug abusers, compared to listening to a control piece of music. Results showed that listening to one’s own performance had a positive influence on several self-reported psychological variables, but that these observed effects were largely dependent upon context.

Of primary interest was the finding that listening to self-made music had a positive influence on internal locus of control, and desire to engage in sports activities. Also, we found an influence on mood in relation to the participant’s readiness to engage socially. We propose two potential factors contributing to these psychological benefits. Firstly, the physical exertion associated with the musical feedback during Jymmin provides a strong physiological experience associated with the music performance. We suggest that a combination of the high arousal created by the strenuous performance may, in the course of the previous Jymmin session, be interpreted as an emotional arousal, an association that can later when listening to the music again recreate some of the original emotional experience. Secondly, participants strongly recapitulate pleasant social interactions during the Jymmin intervention, as demonstrated by the positive correlation between participants’ mood and their desire to engage in social activities with their former training partners after listening to self-made music (see Figure 3).

These psychological benefits might be of particular therapeutic use in the context of polydrug abusers. Evidence suggests that polydrug abusers suffer from a decreased control belief and struggle to engage in long-lasting prosocial interactions (Hawkins et al., 1986; Beck and Lindenmeyer, 1997; Peters and Wexler, 2005). It is possible that a self-made music piece acts as a “reminder” of an experience in which the individual was in control, thus re-activating positive thoughts. This might be beneficial for building up positive internal beliefs about one’s ability to control their situation, which explains the present finding of an increased internal locus of control after listening to self-made music. This idea is further substantiated by the finding that control belief after listening to a recording of a previous Jymmin session correlated with mood such that the higher the control belief after listening to the recording, the better the mood of the participant (see Figure 4).

Importantly, our data revealed a powerful influence of context on the aforementioned psychological benefits of the physiological and emotional associations formed during this specific form of musical intervention. The fact that self-made music provided a psychological benefit exclusively when it was presented in the first block is an indication of context dependency. When participants were presented with a control music piece first, the psychological benefits of listening to recording of the interactively performed music deteriorated. Playing physical instruments like the drum or the bass is usually already associated with body movements. This may be partly underlying the effect that already passive music listening can lead to substantial rehabilitation effects in stroke patients (Särkämö et al., 2008)—although note that these rehabilitation effects were probably also due to emotional and motivational effects when patients listened to music they enjoyed. It may be that the deterioration of the effect after listening first to other, commercially available music, may be due to an activation of already existing associations with the commercially available music. This might lead to a diminished impetus of the newly formed associations with the own musical performance. Listening to the commercially available music may lead to auditory-motor and a number of extra-musical symbolical associations that probably strongly determine musical meaning (see e.g., Fritz et al., 2013c). Such associations could, for example, include previous scenarios of drug abuse in environments where music was played. It is unclear if participants might have guessed the experimental manipulation and if this might have biased how they assessed the stimuli. However, if this were the case then one would expect that such a bias would influence the assessment of stimuli irrespective of context, that is the sequence in which the conditions (experimental condition, control condition) were presented, which was not the case.

We observed a significant difference in mood in the control condition before listening to the Jymmin music (sequence two) and after listening to Jymmin music (sequence one). This effect on mood measured only in the control condition is rather unclear, and we can only speculate about possible explanations. The increase in mood in the control condition of sequence one (after listening to the Jymmin recording) may correspond to a sustain of mood effects of listening to a recording of Jymmin, similar to effects observed in a previous study where participants actually performed Jymmin (Fritz et al., 2013b). In this previous research it was shown that alterations in mood evoked by Jymmin lasted for a substantial time (more than 15 min) and was therefore probably hormonally mediated (Fritz et al., 2013b). In the current study we did not observe any significant effects on mood after listening to a Jymmin recording (only in relation to the readiness to engage socially, see Figure 3). However, we did observe a descriptive difference between median values after listening to the Jymmin recording and listening to control music. It seems plausible that the lack of statistical significance may be due to the relatively small sample size investigated here (especially when comparing the results to previous studies that measured more participants; Fritz et al., 2013a,b).

The data also show a correlation of positive mood and perceiving the training partners as more likeable and interesting. Positive mood furthermore correlated with the desire to take part in another Jymmin session with the same training partners and the desire to perform general activities with the former training partners. This seems to indicate positive social effects of listening to the previously recorded self-made music.

It is yet unclear if the transience of the psychological benefits of listening to self-made music is exclusive to polydrug abusers. However, it is reasonable to speculate that this may relate to physiological adaptations in the reward system of substance abusers (Koob and Le Moal, 2005), such that the sensitivity of previously formed physiological and emotional musical associations to context (sequence in which conditions were performed in the experiment) observed in the present study might reflect a malfunction in the reward system in polydrug abusers.

A limitation to the present study is that while we chose a similar musical style (drum and bass) for the control condition, and while we selected a different commercially available music piece for every participant to ensure ecological validity, it would have been advantageous to control for tempo and other acoustic features, given that these have a strong influence on for example arousal and mood (Husain et al., 2002). A further limitation is that the beneficial effect of listening to a recording of Jymmin seemed to only be present when the Jymmin recording was presented without previously presenting other control music, which might constitute a potential problem to validate and implement the intervention.

In conclusion, listening to a recording of their own music performance seemed to have a positive influence on self-efficacy (internal locus of control), mood, and the desire to engage in sports activities in polydrug abusers in rehabilitation. However, we also observed a strong influence of context such that the effects of listening to the self-made music were only observed when participants listened to their own performance first. The data furthermore seems to indicate positive social effects, showing a correlation between an influence of the music recording on the participants’ mood and their desire to engage in social activities with their former training partners and how likeable and interesting they were perceived. The context dependence of the observed effects is discussed in terms of other motor and symbolic associations of the commercially available control condition music that may temporarily have overwritten the associations created in the previous musical feedback intervention.

## Conflict of Interest Statement

The Max Planck Society has a pending patent application on the specific combination of musical expression and exercise workout referred to in the manuscript.
